# Daily Physical Activity Patterns: A Window on Cognitive Decline in the Baltimore Longitudinal Study of Aging (BLSA)

**DOI:** 10.1093/geroni/igab046.1721

**Published:** 2021-12-17

**Authors:** Amal Wanigatunga, Fangyu Liu, Hang Wang, Jacek Urbanek, Yang An, Eleanor Simonsick, Susan Resnick, Jennifer Schrack

**Affiliations:** 1 Johns Hopkins Bloomberg School of Public Health, Baltimore, Maryland, United States; 2 Johns Hopkins University, Baltimore, Maryland, United States; 3 Johns Hopkins School of Medicine, Baltimore, Maryland, United States; 4 NIA, Baltimore, Maryland, United States; 5 National Instute on Aging/NIH, Baltimore, Maryland, United States; 6 National Institute on Aging, Baltimore, Maryland, United States

## Abstract

Gradual disengagement from essential daily physical activity (PA) necessary for independent living could signal present or emerging mild cognitive impairment (MCI) or Alzheimer’s disease (AD). We used BLSA data to examine whether PA patterns including: 1) total activity counts/day, 2) minutes/day spent active, and 3) activity fragmentation (reciprocal of the mean active bout length) differs between participants with adjudicated normal cognition (n=498) and MCI/AD diagnoses (n=32). Linear models were used and adjusted for demographics, APOE-e4 status, morbidity, and gait speed. Compared to those with normal cognition, those with MCI/AD had 3.0% higher activity fragmentation (SE=1.1%, p=0.006) but similar mean total activity counts/day (p=0.08) and minutes/day spent active (p=0.19). Results suggest that activity fragmentation may arise as a compensatory strategy in the absence of reduced activity in MCI and early AD and that activity monitoring may be potentially useful for detecting MCI and AD at an earlier stage.

